# The Feasibility and Cost-Effectiveness of Tailored Social Media Advertising to Recruit Sexual and Gender Minority Adolescents in the Southern United States: Online Survey Study

**DOI:** 10.2196/95006

**Published:** 2026-08-31

**Authors:** Danielle Lambert, Nicole Luisi

**Affiliations:** 1 Department of Epidemiology & Biostatistics, College of Public Health, University of Georgia, 101 Buck Road, B.S. Miller Hall, Athens, Georgia, 30602, Georgia; 2 Department of Epidemiology, Rollins School of Public Health, Emory University, Atlanta, GA, United States

**Keywords:** sexual and gender minorities, adolescents, online recruitment, social media, social media recruitment, costs and cost analysis, web-based survey

## Abstract

This study assesses the feasibility and costs of reaching, screening, and enrolling sexual and gender minority adolescents aged 13-17 years in the Southern United States in an online mixed method study, with a focus on state-level differences in recruitment and expense.

## Introduction

An increasing number of studies in recent years have evaluated the success and cost efficiency of using social media-based recruitment compared to nondigital strategies to enroll participants [1], though findings specific to social media recruitment among hard-to-reach, minority, and adolescent populations are still limited [2]. Available scientific literature about the success of specific recruitment strategies and potential cost differences by state are even more limited [3], especially for research focused on highly stigmatized and politicized topics or issues. The aim of this analysis was to evaluate the feasibility and costs of recruiting sexual and gender minority adolescents in the 8 Southern US states, a region noted for greater stigma around sexual and gender identity and increasing anti-LGBTQIA+ (lesbian, gay, bisexual, transgender, queer, intersex, and asexual) legislation [4-6].

## Methods

### Study Population and Setting

This study primarily focused on assessing the prevalence of and lived experiences around digital dating violence, and co-occurring in-person teen dating violence, among sexual minority and gender diverse adolescents in the Southern United States. Recruitment was concentrated on 8 southeastern states due to regional similarities in internal and external stigma and discrimination, religiosity, increasingly restrictive policies around health care access and sexual and gender identity, and barriers to care. To be eligible for participation, respondents must: (1) be 13‐17 years of age; (2) live in Alabama, Florida, Georgia, Louisiana, Mississippi, North Carolina, South Carolina, or Tennessee; (3) identify as LGBTQIA+; (4) report at least 1 prior sexual or romantic partner; and (5) be comfortable reading and writing in English.

### Ethical Considerations

This study was approved by the University of Georgia’s institutional review board prior to any data collection (PROJECT00003480).

### Online Recruitment Methods

From July 2021 to April 2022, we used a combination of 9 different still image and video-based advertisements on Facebook and Instagram platforms to recruit adolescents for study participation. In total, we ran 80 different combinations of paid advertisement sets, all but one were tailored to a specific state (ie,~10 advertisements per state with 9 different visuals). Users clicked or swiped up on the advertisements to be redirected to the study screener, at which point they could read more about the study procedures, self-screen, and provide demographic and contact information for our study team to verify their identity and complete enrollment. Within 3‐5 days, study staff used multiple authentication measures to verify that each eligible respondent was a unique, real individual meeting our eligibility criteria. Duplicate and fraudulent responses or those unable to be verified were unable to proceed with study enrollment. Validated respondents were emailed an individualized, single-use link to complete the online survey assessment. At the end of the survey, respondents were prompted to confirm their enrollment email and reverify their identity to receive the $20 incentive. Adolescents who completed the assessment could also opt-in to a future Zoom-based qualitative interview with an additional US $30 incentive.

## Results

### Feasibility of Social Media Advertising for Adolescent Recruitment

**Figure 1.**
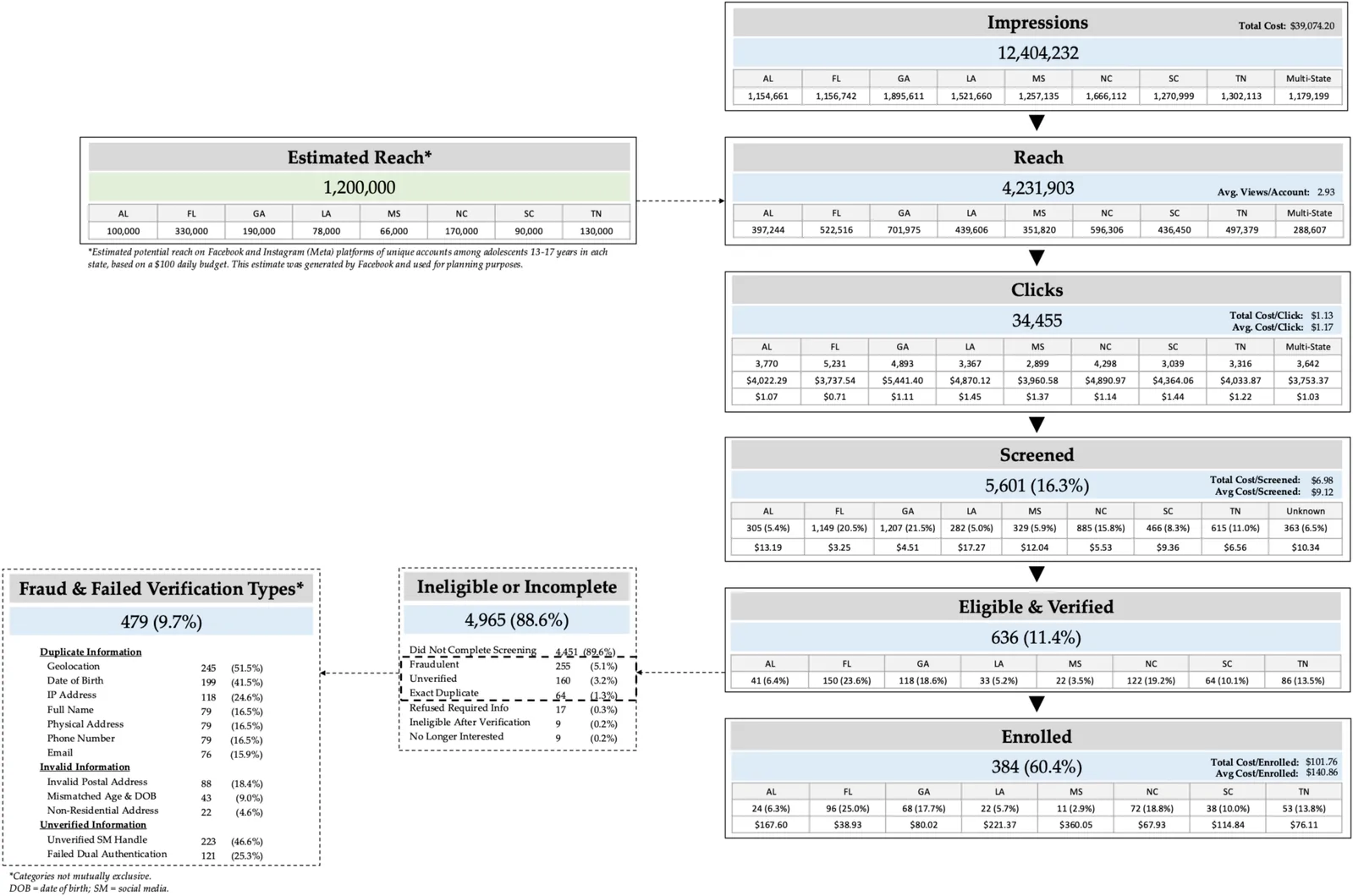
STROBE (Strengthening the Reporting of Observational Studies in Epidemiology) diagram depicting the feasibility of using tailored social media advertising to recruit and enroll LGBTQIA+ (lesbian, gay, bisexual, transgender, queer, intersex, and asexual) adolescents, stratified by state, in the Southern United States, July 2021–April 2022. AL: Alabama; FL: Florida; GA: Georgia; LA: Louisiana; MS: Mississippi; NC: North Carolina; SC: South Carolina; TN: Tennessee.

The tailored advertisement sets targeting adolescents 13‐17 years in 8 southern states resulted in 12.4 million advertisement views seen by 4.2 million unique social media accounts and converted to 34,455 clicks redirecting to the study screener (Figure 1). By state, advertisements in Georgia resulted in the greatest number of impressions (n=1,895,611) and unique accounts reached (n=701,975), while Alabama had the fewest impressions (n=1,154,661) and Mississippi had the fewest accounts reached (n=351,820). The greatest number of conversions to the screener was in Florida (n=5231), in comparison with almost half that number in Mississippi (n=2899). Among screeners, 41.5% (n=2356) were a result of advertisement clicks in Georgia (n=1207) and Florida (n=1149) (21.5% and 20.5%, respectively). The fewest screeners resulted from advertisements in Louisiana (n=282) and Alabama (n=305) (5.0% and 5.4%, respectively). The prevalence of fraudulent and duplicate responses, and the authentication methods used to identify them, are summarized in Figure 1.

### Costs Associated With Advertising, Screening, and Enrollment

**Figure 2.**
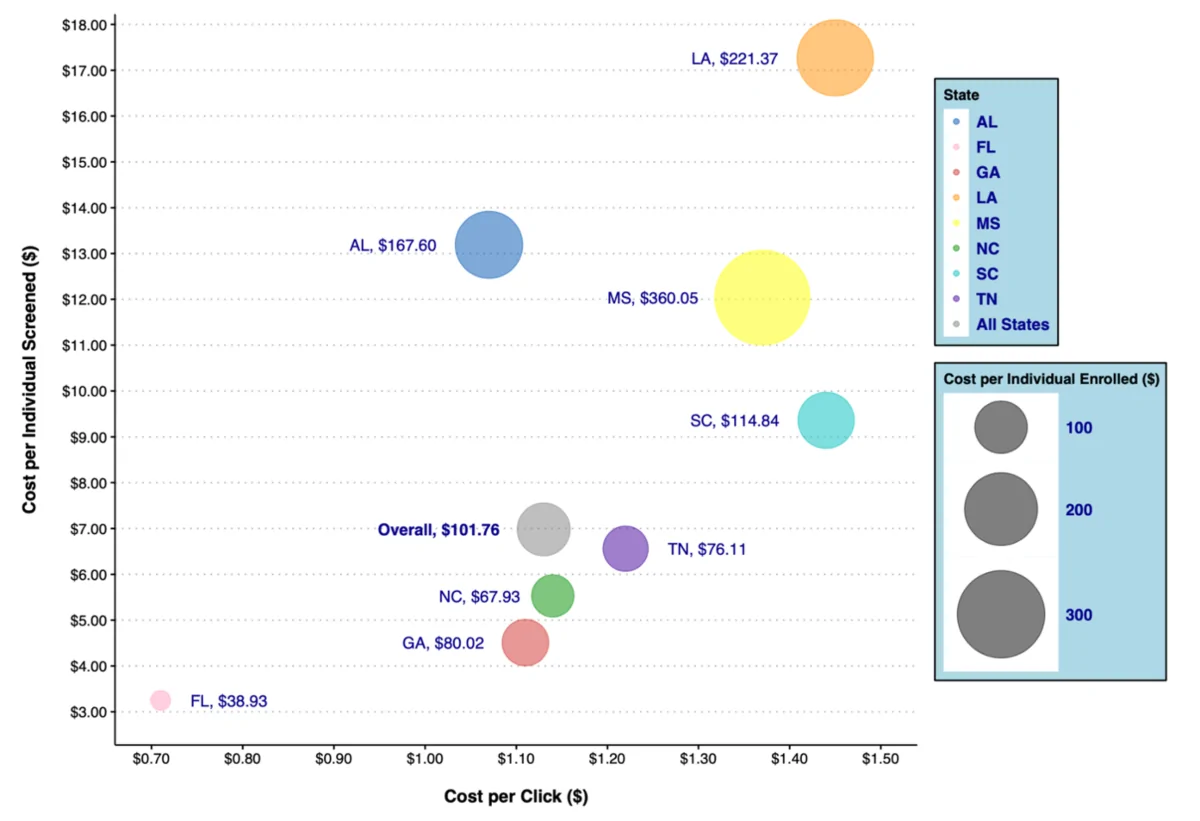
The cost (in US $) of advertising to, screening, and enrolling LGBTQIA+ (lesbian, gay, bisexual, transgender, queer, intersex, and asexual) youth in the Southern United States for an online mixed methods study, stratified by state of residence, July 2021–April 2022. AL: Alabama; FL: Florida; GA: Georgia; LA: Louisiana; MS: Mississippi; NC: North Carolina; SC: South Carolina; TN: Tennessee.

We spent US $39,074.20 on social media advertising, with an overall cost per click of US $1.13 (Figure 2) ranging from US $0.71 in Georgia to US $1.45 in Louisiana. The overall cost per completed screener was US $6.98, ranging from US $3.25 in Florida to US $17.27 in Louisiana. The final cost per enrolled adolescent was lowest in Florida (US $38.93) and highest in Mississippi (US $360.05), with an overall cost per enrolled adolescent of US $101.76.

### Sociodemographics of Enrolled Sample

A diverse sample of 384 adolescents participated in the study, with respondents from all 8 states ranging in age from 13 to 17 (mean 16.1, SD 1.0) years. The sample was 45.6% (n=175) female, 52.6% (n=202) non-Hispanic white, and 38.0% (n=146) bisexual. In total, 33.3% (n=128) of enrolled adolescents were nonbinary and 13.0% (n=50) were transgender.

## Discussion

Using social media to recruit a diverse sample of sexual and gender minority adolescents from the Southern US states was a successful and cost-effective strategy, mirroring past studies [1,3,7,8], though differences in reach and cost were noted by the states’ estimated adolescent population, urbanicity, and legislative landscape. The states with the highest cost per screened and enrolled adolescent (Mississippi: $12.04, $360.05; Louisiana: $17.27, $221.37; Alabama: $13.19, $167.60; South Carolina: $9.36, $114.84, respectively) were the states with the smallest estimated population of adolescents 14‐17 years, as of July 1, 2021 (Mississippi: n=168,031; Louisiana: n=240,240; Alabama: n=265,054; South Carolina: n=267,163) [9]. Conversely, Florida, which had the lowest cost per screened and enrolled adolescent ($3.25 and $38.93, respectively), had the highest estimated adolescent population (n=1,015,069) [9]. States with the most expensive recruitment and enrollment costs were also those with a greater number of anti-LGBTQIA+ bills introduced during the 2021 and 2022 legislative sessions (n=101; Tennessee: n=28, South Carolina: n=19, Mississippi: n=11, Alabama: n=11) [4,5], which may have influenced the adolescents’ willingness to participate in research specific to their sexual and gender identity due to concerns of confidentiality in the current sociopolitical climate. The states with the lowest cost per screened and enrolled youth (Florida, North Carolina, Tennessee, and Georgia) were states with some of the largest US metropolitan statistical areas (MSAs), including the Miami, Charlotte, Nashville, and Atlanta MSAs [10]. These findings indicate that recruitment strategies should account for potential state-level factors, and may require additional allocation of funding in states with smaller population estimates, fewer MSAs, and more restrictive policies related to the research topic.

## Abbreviations

LGBTQIA+: Lesbian, gay, bisexual, transgender, queer, intersex, and asexual
MSA: metropolitan statistical area
